# Potential of Nucleic Acid Receptor Ligands to Improve Vaccination Efficacy against the Filarial Nematode *Litomosoides sigmodontis*

**DOI:** 10.3390/vaccines11050966

**Published:** 2023-05-10

**Authors:** Johanna F. Scheunemann, Frederic Risch, Julia J. Reichwald, Benjamin Lenz, Anna-Lena Neumann, Stephan Garbe, Stefan J. Frohberger, Marianne Koschel, Jesuthas Ajendra, Maximilian Rothe, Eicke Latz, Christoph Coch, Gunther Hartmann, Beatrix Schumak, Achim Hoerauf, Marc P. Hübner

**Affiliations:** 1 Institute for Medical Microbiology, Immunology and Parasitology, University Hospital Bonn, 53127 Bonn, Germanyrisch.frederic@googlemail.com (F.R.); jaje@uni-bonn.de (J.A.); hoerauf@uni-bonn.de (A.H.); 2 Clinic for Radiotherapy and Radiation Oncology, University Hospital Bonn, 53127 Bonn, Germany; 3 Institute for Innate Immunity, University Hospital Bonn, 53127 Bonn, Germany; 4 Institute for Clinical Chemistry and Clinical Pharmacology, University Hospital Bonn, 53127 Bonn, Germany; 5 Nextevidence GmbH, 81541 Munich, Germany; 6 German Center for Infection Research (DZIF), Partner Site Bonn-Cologne, 53127 Bonn, Germany

**Keywords:** filariae, *Litomosoides sigmodontis*, 3pRNA, poly(I:C), vaccination, nucleic acid receptor, helminth

## Abstract

More than two-hundred-million people are infected with filariae worldwide. However, there is no vaccine available that confers long-lasting protection against filarial infections. Previous studies indicated that vaccination with irradiated infective L3 larvae reduces the worm load. This present study investigated whether the additional activation of cytosolic nucleic acid receptors as an adjuvant improves the efficacy of vaccination with irradiated L3 larvae of the rodent filaria *Litomosoides sigmodontis* with the aim of identifying novel vaccination strategies for filarial infections. Subcutaneous injection of irradiated L3 larvae in combination with poly(I:C) or 3pRNA resulted in neutrophil recruitment to the skin, accompanied by higher IP-10/CXCL10 and IFN-β RNA levels. To investigate the impact on parasite clearance, BALB/c mice received three subcutaneous injections in 2-week intervals with irradiated L3 larvae in combination with poly(I:C) or 3pRNA prior to the challenge infection. Vaccination with irradiated L3 larvae in combination with poly(I:C) or 3pRNA led to a markedly greater reduction in adult-worm counts by 73% and 57%, respectively, compared to the immunization with irradiated L3 larvae alone (45%). In conclusion, activation of nucleic acid-sensing immune receptors boosts the protective immune response against *L. sigmodontis* and nucleic acid-receptor agonists as vaccine adjuvants represent a promising novel strategy to improve the efficacy of vaccines against filariae and potentially other helminths.

## 1. Introduction

Helminth infections affect more than 1 billion people and put a high socioeconomic burden on endemic countries [1]. Filariae are extraintestinal helminths, which can cause debilitating diseases such as lymphatic filariasis (elephantiasis) and onchocerciasis (river blindness). Currently, there are 859 million and 218 million people living in areas of ongoing transmission of these diseases, respectively [2]. The elimination of both diseases by 2030, at least in a considerable percentage of countries, was stated as a goal of the World Health Organization (WHO) [2]. The control of both diseases is mainly mediated by annual mass drug administrations of drugs that temporarily inhibit filarial embryogenesis and the transmission of the disease, but do not kill the adult filariae [3,4]. To support elimination, the development of a potent prophylactic or therapeutic vaccine would be a powerful tool. However, currently, there is no vaccination available for any human helminth infection and potential vaccine candidates are providing only limited protection [5,6]. In the case of *Schistosoma* spp., four vaccine candidates, including the *Schistosoma haematobium* glutathione S-transferase, *Schistosoma mansoni* fatty acid-binding protein, *S. mansoni* tetraspanin, and *S. mansoni* calpain, are being evaluated in clinical trials with aluminum-based adjuvants [7]. Initial phase I/II clinical trials showed all vaccine candidates to be well tolerated and immunogenic; however, the efficacy in humans is either still unclear or suboptimal (*S. haematobium* glutathione S-transferase) [7]. For the nematode *Onchocerca volvulus*, there has been progress using recombinant *O. volvulus* antigen, i.e., *Ov*-103 and *Ov*-RAL-2, which induced protections of up to 64% in combination with alum-based adjuvants in a murine model [8,9]. Further, coadministration of *Ov-103* and *Ov-*RAL-2 in the bovine model utilizing infections with *Onchocerca ochengi* led to a significant decrease in the rate of infection and development of microfilaridermia in cows that were infected via natural exposure [10]. Similarly, vaccinations with a fusion protein of the orthologous proteins from *Brugia malayi* (*Bm-*103 and *Bm-*RAL-2) coadministered with alum resulted in a significant worm reduction (61%) and reduced the embryogenesis of the remaining worms in a gerbil model [11]. The putative vaccine against *O. volvulus* is currently in late preclinical development. For filarial infections in general, one of the best efficacies is obtained when irradiated L3 larvae are used for vaccinations, which can be used to study protective immune responses and explore novel vaccination approaches [12,13].

In this project, we used the *Litomosoides sigmodontis* murine model to identify new vaccination strategies. *L. sigmodontis* establishes chronic infections in susceptible BALB/c mice and triggers immune responses that resemble those of human filarial infections [14,15,16]. Previous studies showed that a triple immunization with irradiated *L. sigmodontis* L3 larvae every week resulted in the generation of parasite-specific IgG1 antibodies and a reduction in the adult-worm burden following a challenge infection of around 50% [12,13,17,18]. 

One of the challenges to developing vaccines against helminth infections is the modulation of the host’s immune system by the parasite. During helminth infections, the host’s immune system is strongly regulated by the parasite in order to facilitate its survival in the host [19,20]. Type 2-associated immune responses, including the expansion of eosinophils, IL-4/IL-13 activated macrophages, and ILC2s as well as increased production of type 2 cytokines such as IL-4 and IL-5, have been linked to protection [16,21,22]; however, a type 1/type 2 balanced immune response also appears to be crucial for parasite elimination [6,23,24,25]. To overcome those protective immune responses, helminths release immunomodulatory molecules and establish a regulatory, anti-inflammatory immune milieu over time [16,19,20,26,27,28]. 

So far, a possible contribution of nucleic acid immunity in the context of helminth infection has not been addressed. Nucleic acid immunity is best studied in the context of viral infections where it triggers dominant type 1-associated immune responses [29]. Different RNA (e.g., RIG-I, MDA5; TLR7/8) and DNA (e.g., via STING; TLR9) sensors in the cytosol or endosome of eukaryotic cells activate downstream cascades that involve the induction of a type I interferon (IFN) response which provides innate antiviral immunity [27,30]. Nucleic acid receptors agonists are also promising vaccine adjuvants that are currently developed for vaccines against cancer and viral infections [31,32,33,34]. However, a possible impact of type I IFN responses on filarial infections and the impact of nucleic acid receptor ligands on filarial vaccination efficacy is not known yet.

In this study, we show that the use of nucleic acid receptor agonists as a vaccine adjuvant enhances local immune responses, including the production of type I IFN during immunization, which results in increased parasite-specific immune responses and enhanced worm clearance after infectious challenge. Consequently, we present the use of the cytosolic RNA receptors agonists as a novel strategy to improve filarial vaccination efficacy.

## 2. Materials and Methods

### 2.1. Animals and Infection

Six-week-old female BALB/c mice and eight-week-old *Meriones unguiculatus* were purchased from Janvier Labs, Saint-Berthevin, France. All animals were housed in individually ventilated cages within the animal facility at the IMMIP, University Hospital Bonn, with unlimited access to food and water and a 12 h day/night cycle.

Mice and *Meriones unguiculatus* were naturally infected with *L. sigmodontis* via exposure to *Ornithonyssus bacoti* mites containing the infective L3 larvae for 24 h as previously described [35]. 

### 2.2. Parasite Recovery 

To generate *L. sigmodontis* L3 larvae, *M. unguiculatus* were euthanized 5 days after the infection with an overdose of isoflurane (AbbVie, Wiesbaden, Germany), and the L3 larvae were isolated by pleural lavage with 25 mL warm Advanced-RPMI medium (Thermo Fisher Scientific, Waltham, MA, USA). 

To quantify the adult-worm burden, mice were euthanized 63 days after the infection with an overdose of isoflurane (AbbVie, Wiesbaden, Germany), and the adult worms were isolated by pleural lavage with 8–10 mL cold PBS (Thermo Fisher Scientific, Waltham, MA, USA). The isolated worms were quantified, and the gender of the adult worms was determined. 

For microfilariae quantification, 50 µL of peripheral blood were drawn from the facial vein via a puncture with a 4 mm animal lancet (Braintree Scientific, Braintree, MA, USA) to EDTA tubes and incubated with 950 μL 1× red blood cell lysis buffer (Thermo Fisher Scientific Inc., Waltham, Massachusetts, USA) for 10 min at room temperature (RT). The blood was then centrifuged (400× *g*, 5 min, RT) and the supernatant was discarded. Microfilariae in the pellet were then counted via light microscopy.

### 2.3. Agonist Injection and Immunization Protocol

Twenty µg/mouse poly(I:C) [36,37,38,39] (HMW) VacciGrade^TM^ (Invivogen, San Diego, CA, USA) and 20 µg/mouse 3pRNA [40,41] (synthesized by AG Hartmann, University Hospital Bonn, Bonn, Germany) were formulated with in vivo-jetPEI^®^ (Polyplus-transfection SA, New York City, NY, USA) according to the manufacturer’s protocol at an N/P ratio of 8.

Attenuation of L3 larvae by irradiation was performed at the Department of Radiation Oncology, University Hospital Bonn, Germany. Radiation was performed using a TrueBeam STx^®^ (Varian Medical Systems, Palo Alto, CA, USA). The photon energy of the radiation source was 10 MeV with a dose rate of 24 Gy/min. L3s were irradiated with 450 Gy [42] and applied in consecutive fractions without break with 100 Gy per fraction in a tissue equivalent RW3-Plasticphantom at a depth of 23 mm (dose maximum). 

**Figure 1:**
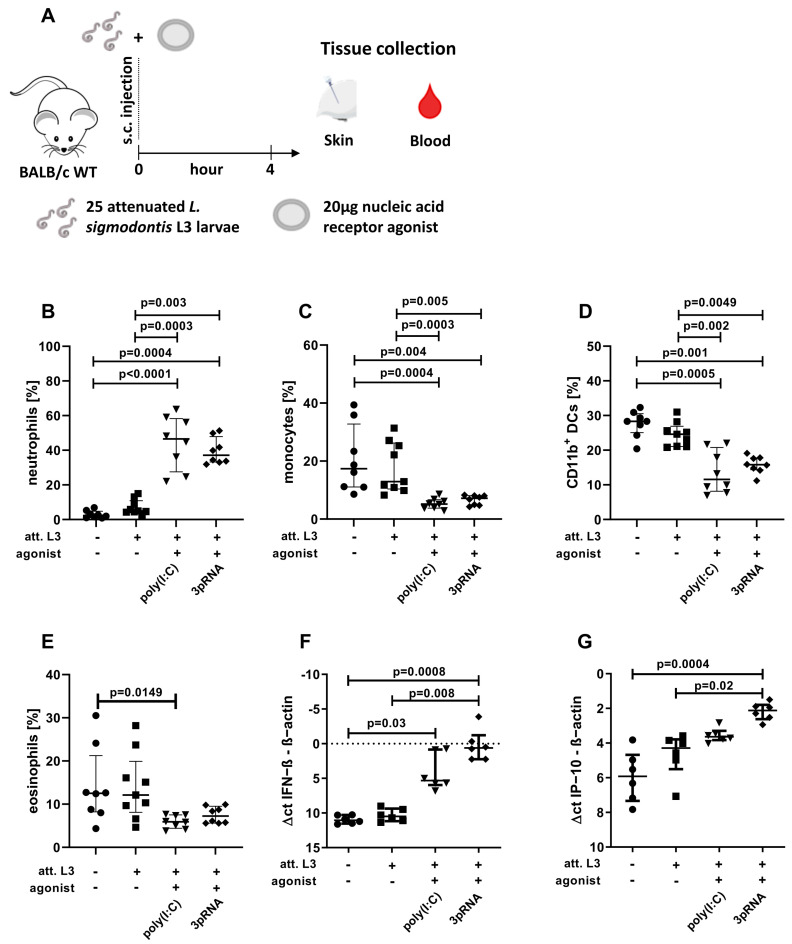
Enhanced local immune activation after injection of poly(I:C) or 3pRNA. (**A**–**G**) Mice were injected subcutaneously with attenuated (att.) *L. sigmodontis* L3 larvae with or without 3pRNA and poly(I:C) and the skin was analyzed four hours after injection. (**B**–**G**) Black circles indicate naïve animals, black squares animals receiving att. *L. sigmodontis* L3 larvae, black triangles animals receiving att. *L. sigmodontis* L3 larvae plus poly(I:C), black diamonds animals receiving att. *L. sigmodontis* L3 larvae plus 3pRNA. (**A**) Experimental setup. (**B**–**E**) Skin cells were analyzed by flow cytometry. Frequency of (**B**) neutrophils (CD45^+^CD11b^+^Ly6G^+^), (**C**) monocytes (CD45^+^CD11b^+^Ly6C^+^Ly6G^−^), (**D**) CD11b^+^ DCs (CD45^+^CD11b^+^Ly6C^−^Ly6G^−^CD11c^+^SiglecF^−^), and (**E**) eosinophils (CD45^+^CD11b^+^SiglecF^+^) among CD45^+^ cells. (**F**,**G**) Skin samples were analyzed by RT-PCR. The Δct values of (**F**) IFN−β expression and (**G**) IP−10/CXCL10 expression compared to β−actin levels in the corresponding sample. (**B**–**G**) Error bars show the median with IQR. Data were statistically analyzed by Kruskal–Wallis with Dunn’s post hoc test. (**B**–**E**) Data from one experiment, *n* = 8. (**F**,**G**) representative for three experiments with *n* = 5–6.

**Figure 2:**
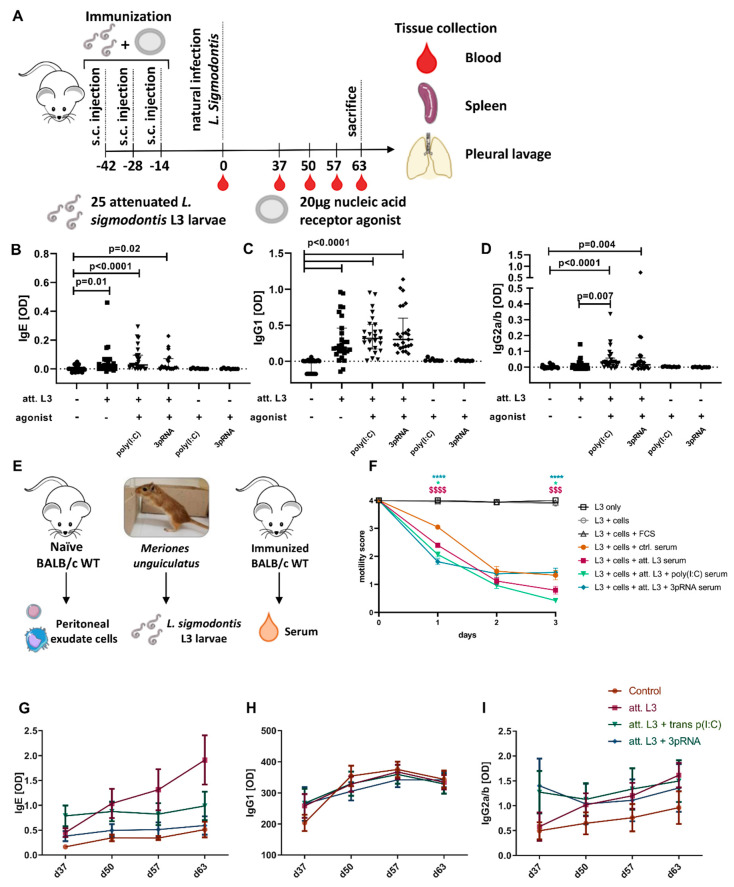
Immunization with adjuvants enhances functional parasite-specific antibody responses. (**A**–**D**) Mice were immunized three times in two−week intervals by subcutaneous injection of attenuated (att.) *L. sigmodontis* L3 larvae in combination with poly(I:C) or 3pRNA. (**A**) Experimental setup. (**B**–**D**) *L. sigmodontis*-specific (**B**) IgE, (**C**) IgG1, and (**D**) IgG2a/b serum antibody levels two weeks after the last injection and before challenge infection. Black circles indicate naïve animals, as well as animals that received either poly(I:C) or 3pRNA alone, black squares indicate animals receiving att. *L. sigmodontis* L3 larvae, black triangles animals receiving att. *L. sigmodontis* L3 larvae plus poly(I:C), black diamonds animals receiving att. *L. sigmodontis* L3 larvae plus 3pRNA. (**E**) Experimental setup of an ADCC assay that was performed using cocultures with a serum of immunized BALB/c mice as well as *L. sigmodontis* L3 larvae and naïve peritoneal exudate cells. The motility of individual larvae shown in (**F**) was assessed for three days by the following score: 4: fast and continuous movement, 3: slower but continuous movement, 2: slower and discontinuous movement, 1: sporadic movement limited to the ends, 0: no movement. (**G**–**I**) Immunized mice were naturally infected with *L. sigmodontis* two weeks after the last immunization injection. Serum was collected 37, 50, 57, and 63 days after infection and analyzed by ELISA for *L. sigmodontis*-specific (**G**) IgE, (**H**) IgG1, and (**I**) IgG2a/b antibodies. (**B**–**D**) Data shown as median with IQR. Statistical analysis using Kruskal–Wallis with Dunn’s post hoc test with *n* = 8 − 28. Data from untreated mice and groups receiving att. L3 larvae with or without agonist were pooled from 3 individual experiments. Data from groups that only received agonist are from one experiment. (**F**) Data presented as mean ± SEM and was statistically analyzed by a 2-way ANOVA with Bonferroni’s multiple comparison test. The ^$$$^ *p* < 0.001, ^$$$$^ *p* < 0.0001: comparison of L3 + att. L3 serum to L3 + ctrl. serum. * *p* < 0.05, **** *p* < 0.0001: comparison of groups including agonist treatment to L3 + att. L3 serum. Data from L3 + cells + FCS pooled from two independent experiments. Other data were pooled from three individual experiments. For all groups, *n* = 49–72 larvae. (**G**–**I**) Data from one experiment presented as mean ± SEM (*n* = 6–10) and was analyzed by a 2-way ANOVA with Tukey’s multiple-comparison test for differences at each time point.

**Figure 3:**
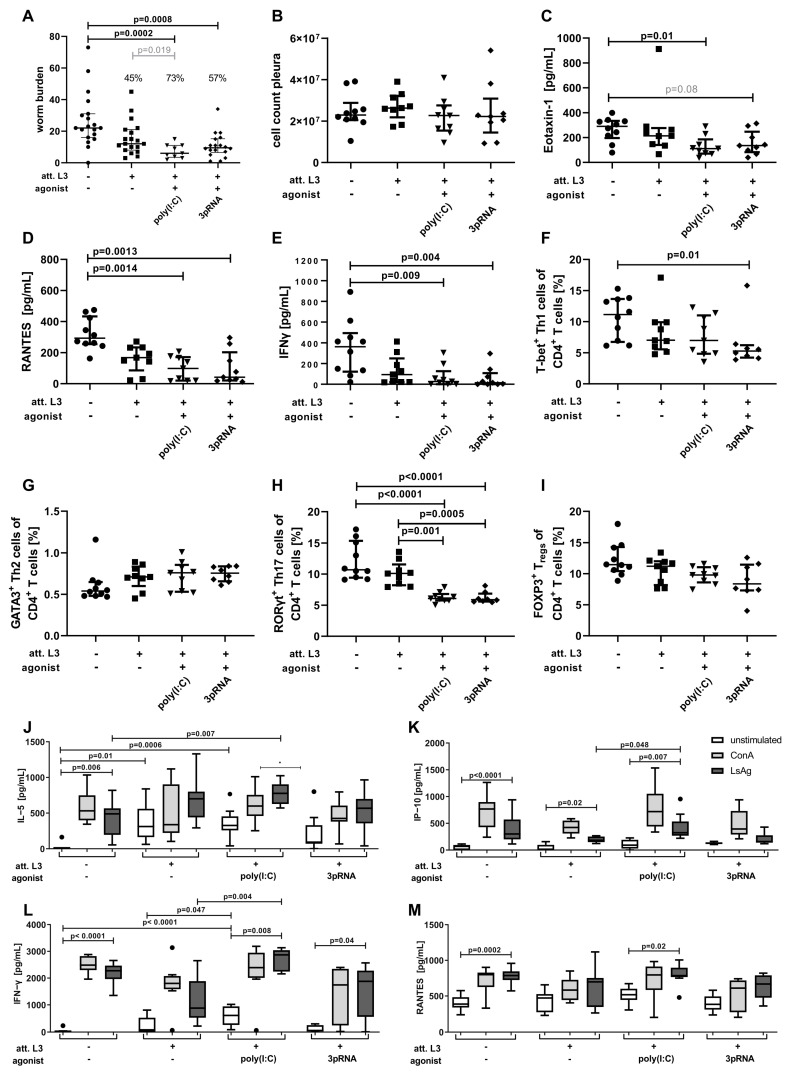
Immunization with poly(I:C) or 3pRNA as an adjuvant significantly reduces the worm burden following challenge infection in mice. (**A**–**M**) Mice were immunized three times in two-week intervals by subcutaneous injection of attenuated (att.) *L. sigmodontis* L3 larvae in combination with poly(I:C) or 3pRNA. Two weeks after the last injection, the mice were naturally infected with *L. sigmodontis* for 63 days. (**A**–**I**) Black circles indicate naïve animals, black squares animals receiving att. *L. sigmodontis* L3 larvae, black triangles animals receiving att. *L. sigmodontis* L3 larvae plus poly(I:C), black diamonds animals receiving att. *L. sigmodontis* L3 larvae plus 3pRNA. (**A**) Quantification of adult worms and (**B**) total cell count in the pleural cavity. Quantification of (**C**) Eotaxin-1/CCL11, (**D**) RANTES/CCL5 and (**E**) IFN−γ in the pleura lavage by ELISA. Frequency of (**F**) Th1 (CD3^+^CD4^+^CD8^−^FOXP3^−^T−bet^+^), (**G**) Th2 (CD3^+^CD4^+^CD8^−^FOXP3^−^T−bet^−^GATA3^+^), (**H**) Th17 (CD3^+^CD4^+^CD8^−^FOXP3^−^T−bet^−^GATA3^−^RORγt^+^) and (**I**) Treg cells (CD3^+^CD4^+^CD8^−^FOXP3^+^) in the spleen quantified by flow cytometry analysis. (**J**) IL−5, (**K**) IP−10, (**L**) IFN−γ, (**M**) RANTES/CCL5 levels determined via ELISA of splenocytes that were restimulated with Concanavalin A (ConA, grey bars), *L. sigmodontis* adult-worm extract (LsAg, black bars) or left unstimulated (white bars). (**A**–**I**) Data shown as median with IQR. (**J**–**M**) Data shown as box and whiskers blot. (**A**–**M**) Data were statistically analyzed by Kruskal–Wallis with Dunn’s post hoc test. (**A**) Grey number indicates *p* value as assessed by direct comparison (Mann-Whitney U test). (**C**) Grey number indicates p value between 0.05 and 0.1. (**A**) Pooled data from two individual experiments. (**B**) Data from one experiment (**C**–**M**) and representative for two individual experiments with *n* = 6–20.

Then, 25 irradiated L3s [42] and agonists were injected in separate syringes with a volume of 50 µL each, right after the other in the same injection area at the hind leg for a skin analysis (Figure 1) or in the neck for full vaccination (Figure 2 and Figure 3).

For the skin analysis (Figure 1), the right hind leg of the female BALB/c mice was shaved one day before the experiment. Larvae and agonists were then injected subcutaneously, as mentioned above, and the injection area was marked with a pen. Four hours later, the mice were euthanized, and the marked area was excised and further processed (see ‘Organ preparation—Skin’).

In the vaccination experiments, mice were subcutaneously injected in the neck with 25 L3 larvae attenuated by 450 Gy radiation (‘att.’) with or without an agonist three times in two-week intervals. Two weeks after the last immunization, blood was drawn, and the mice were naturally infected (day 0). Further, blood was drawn on days 50, 57, and 63 and ex vivo analysis was performed on day 63 after the infection.

### 2.4. Organ Preparation

#### 2.4.1. Blood

Blood for serum was collected from the facial vein of animals via a puncture with a 4 mm animal lancet (Braintree Scientific, Braintree, MA, USA) and stored at RT. The clotted samples were centrifuged (6000× *g*, 5 min, RT) and the serum was stored at −20 °C until analysis.

#### 2.4.2. Skin

Four hours after the injection of L3 larvae and/or agonists, the skin (approx. 1 cm^2^) was excised post mortem. Half of the skin was stored in 700 µL Trizol (QIAGEN, Hilden, Germany) at −20 °C until RNA isolation. The other half was minced and incubated at 37 °C on a shaker (350 rpm) for 75 min in an RPMI medium (Life technologies Corporation, Grand Island, NY, USA), supplemented with 10% FCS (PAN Biotech, Aidenbach, Germany), 1% Penicillin (10,000 units/mL)/Streptomycin (10 mg/mL) (Life technologies Corporation, Grand Island, NY, USA), 2 mM L-Glutamine (Life technologies Corporation, Grand Island, NY USA), 0.25 mg/mL Liberase TL (Hoffmann-La Roche Ltd., Basel, Switzerland), and 0.5 mg/mL DNase I (Thermo Fisher Scientific, Waltham, MA, USA). The reaction was stopped with RPMI medium supplemented with 10 mM EDTA (Carl Roth, Karlsruhe, Germany) and 2% FCS. Cells were passed through a 70 µm cell strainer (Miltenyi Biotec, Bergisch Gladbach, Germany), centrifuged at 400× *g* for 5 min at 4 °C and taken up in MACS buffer (PBS, 1% FCS (PAN Biotech, Aidenbach, Germany), 2 mM EDTA (Carl Roth, Karlsruhe, Germany)). Cells were then counted with a CasyR TT Cell Counter + Analyser System (Schärfe Systems, Reutlingen, Germany), and 1 × 10^6^ cells were analyzed by flow cytometry.

#### 2.4.3. Pleura

The pleural lavage was performed with 8–10 mL cold PBS. The first mL was collected and centrifuged at 400× *g*, 5 min at 4 °C. The obtained supernatant was stored at −20 °C for ELISA analysis, the cell pellet was pooled with the remaining lavage fraction. Red blood cell lysis was performed with RBC buffer according to the manufacturer’s protocol (Thermo Fisher Scientific Inc., Waltham, MA, USA). Cells were washed with MACS buffer, counted with a CasyR TT Cell Counter + Analyser System (Schärfe Systems, Reutlingen, Germany), and 1 × 10^6^ cells were analyzed by flow cytometry.

#### 2.4.4. Spleen

Isolated spleens were perfused with 0.5 mg/mL collagenase VIII buffer (Roche, Basel, Switzerland), minced, and incubated at 37 °C for 30 min on a shaker with 350 rpm. MACS buffer was added, and a single-cell suspension was generated by passing the cells through a 70 µm metal strainer. The cells were centrifuged at 400× *g* for 5 min at 4 °C. RBC lysis was performed according to the manufacturer’s protocol (Thermo Fisher Scientific Inc., Waltham, MA, USA). Afterward, the cells were washed with MACS buffer and taken up in RPMI medium (Life technologies Corporation, Grand Island, NY, USA) supplemented with 10% FCS (PAN Biotech, Aidenbach, Germany), 1% Penicillin (10,000 units/mL)/Streptomycin (10 mg/mL) (Life technologies Corporation, Grand Island, NY, USA) and 2 mM L-Glutamine (Life technologies Corporation, Grand Island, NY, USA). Cells were then counted with a CasyR TT Cell Counter + Analyser System (Schärfe Systems, Reutlingen, Germany), and 1 × 10^6^ cells were analyzed by flow cytometry. For ELISA analysis, 2 × 10^6^ cells/mL were plated and stimulated with 2.5 µg/mL Concanavalin A (ConA, Sigma-Aldrich, St. Louis, MO, USA) or 25 µg/mL crude *L. sigmodontis* adult-worm extract (LsAg) for 72 h in 48-well plates. The supernatant was stored at −20 °C until further analysis.

For LsAg preparation, freshly isolated adult worms were rinsed in sterile PBS before being mechanically homogenized under sterile conditions. Insoluble material was removed by centrifugation at 400× *g* for 10 min at 4 °C. The protein concentrations of crude extracts were determined using the Advanced Protein Assay (Cytoskeleton, Denver, USA).

### 2.5. Determination of Parasite-Specific Antibodies

Plates were coated with 20 µg/mL LsAg diluted in PBS overnight (o/n) at 4 °C. Plates were washed with PBS containing 0.5% Tween^®^ 20 (Merck KGaA, Darmstadt, Germany) and blocked for 2 h at RT with PBS containing 1% bovine serum albumin (BSA) (PAA Laboratories, Cölbe, Germany). Sera from day 0, isolated from the blood that was drawn after the immunization and prior to the challenge infection, were diluted 1:50, sera from following time points after challenge infection were diluted 1:1000 in 1% BSA/PBS and incubated o/n at 4 °C. Plates were washed and biotinylated murine IgE, IgG1 or IgG2a/b antibodies (BD Biosciences San Jose, CA, USA), at a dilution of 1:400 in 1% BSA/PBS, were added and incubated for 2 h at RT on a shaker. After washing, streptavidin-HRP (Thermo Fisher Scientific Inc., Waltham, MA, USA) was added for 30 min at RT on a shaker. After washing, TMB (Thermo Fisher Scientific Inc., Waltham, MA, USA) was added. Upon coloration, the reaction was stopped with 2 M H_2_SO_4_ (Carl Roth GmbH + Co. KG, Karlsruhe, Germany). Reading was performed at 450 nm and 570 nm wavelengths using a SpectraMax190 (Molecular Devices, San Jose, CA, USA) with Soft Max Pro 7 software (Molecular Devices, San Jose, CA, USA).

### 2.6. In Vitro Motility Assay

The assay was adapted from Veerapathran et al. [43]. L3 larvae were recovered by pleural lavage from *M. unguiculatus* 5 days after natural *L. sigmodontis* infection. Peritoneal cells were isolated from naïve BALB/c WT donor mice and 2 × 10^5^ peritoneal cells were cocultured with 10–12 L3 larvae in RPMI-medium (Life technologies Corporation, Grand Island, NY, USA), supplemented with 25% pooled serum drawn from immunized animals immediately prior to the challenge infection (two weeks after the final vaccination). The motility of L3 larvae was scored under the microscope on a daily basis for a total of three days. The following scores were used to assess the motility: 4: fast and continuous movement, 3: slower but continuous movement, 2: slower and discontinuous movement, 1: movement discontinuous and limited to larval ends, 0: no movement observed within 30 s. 

### 2.7. Cytokine Quantification by ELISA

Cytokines were quantified in the first mL of pleural wash and the 72 h splenocyte culture supernatant. IL-5 and IFN-γ were quantified using Invitrogen™ eBioscience™ ELISA Ready-SET-Go!™ (Thermo Fisher Scientific, Waltham, MA, USA). IP-10 (CXCL10), RANTES (CCL5), and Eotaxin 1 (CCL11) were quantified using DuoSet ELISA kits (R&D Systems, Minneapolis, MN, USA). The manufacturers’ protocol was followed. 

The 2 M H_2_SO_4_ (Carl Roth GmbH + Co. KG, Karlsruhe, Germany) served as a stopping solution. Reading was performed at 450 nm and 570 nm wavelength using a SpectraMax190 (Molecular Devices, San Jose, CA, USA) with Soft Max Pro 7 software (Molecular Devices, San Jose, CA, USA).

### 2.8. Flow Cytometric Analysis of Skin, Pleura, and Spleen Cells

The 1 × 10^6^ cells were used per flow cytometry staining. For surface staining, cells were incubated for 20 min with a mastermix prepared in Fc-block (1%FCS/PBS with 0.1% rat IgG (Sigma-Aldrich, St. Louis, MO, USA)). Mastermixes were prepared as combinations of the following antibodies. If not stated otherwise, the antibodies were purchased from BioLegend, San Diego, CA, USA: CD3 (BV510, clone 145-2C11), CD4 (Al700, clone GK1.5), CD8 (PerCP Cy5.5, clone 53-6.7), CD11b (Al700, clone M1/70), CD11c (BV605, clone N418), CD45 (PerCPCy5.5, clone 30-F11), CD86 (Al647, clone GL-1), FOXP3 (PE-Cy7, clone FJK-16s, Thermo Fisher Scientific Inc., Waltham, Massachusetts, USA), GATA3 (Al488, clone 16E10A23), I-Ab (PE-Cy7, clone AF6-120.1), Ly6C (APC-Cy7, clone HK1.4), Ly6G (BV421, clone 1A8), purified RELM-α polyclonal, rabbit, (PeproTech, Inc., Rocky Hill, NJ, USA) combined with goat anti-rabbit Al488 (Invitrogen, Carlsbad, CA, USA), RORγt (PE, AFKJS-9), and T-bet (APC, clone 4B10), SiglecF (PE, clone E50-2440, BD, San Jose, CA, USA). 

For intracellular stainings, cells were incubated in a fixation/permeabilization buffer (Thermo Fisher Scientific Inc., Waltham, Massachusetts, USA) for 20 min at RT. Cells were washed and blocked overnight in Fc-block (1% bovine serum albumin fraction V (BSA) (PAA Laboratories, Cölbe, Germany) in PBS with 1:1000 rat IgG (Sigma-Aldrich, St. Louis, MO, USA)) at 4 °C. The next day, cells were permeabilized with a permeabilization buffer for 20 min at RT (Thermo Fisher Scientific Inc., Waltham, MA, USA) and stained with mastermix containing antibodies for extracellular and intracellular targets for 45 min at 4 °C. After staining, the cells were washed. Data acquisition was performed on a CytoFLEX S (Beckman Coulter, Brea, CA, USA) and analysis with FlowJo^®^ Software V10 (FlowJo, LLC, Ashland, OR, USA). Fluorescence minus one (FMO) controls were used for evaluation.

### 2.9. RNA Isolation

Skin samples stored in 700 µL Trizol were homogenized in a Precellys^®^ 2 mL Soft Tissue Homogenizing Ceramic Beads Tube (Cayman Chemical, Ann Arbor, MI, USA) using the Precellys^®^ 24 machine, program “6000 − 3 × 60 − 120”. The homogenate was incubated at RT for 5 min in a fresh vial and 70 µL of 1-Bromo-3-chloropropane (BCP) (Tokyo Chemical Industry, Tokyo, Japan) was added. The sample was then vortexed and incubated for 2–3 min at RT. After centrifugation for 15 min at 12,000× *g* at 4 °C, 350 µL of the aqueous phase was transferred into a 2 mL reaction tube and placed into QIAcube (QIAGEN, Hilden, Germany) for automated RNA isolation. The animal tissue and cell protocol, including an on-column DNase digest, was followed using the RNeasy^®^ Mini kit (QIAGEN, Hilden, Germany). The 70% ethanol was exchanged with 100% ethanol. 

### 2.10. cDNA and RT-PCR

cDNA was generated from 1 µg RNA with the Omniscript^®^ Reverse Transcription Kit (Qiagen, Hilde, Germany) using oligoDT_12–18_ primer (Thermo Fisher Scientific Inc., Waltham, Massachusetts, USA) and RNaseOUT^TM^ recombinant ribonuclease inhibitor (Thermo Fisher Scientific Inc., Waltham, Massachusetts, USA). The mastermixes were prepared using the HotStarTaq^®^ DNA Polymerase kit (QIAGEN, Hilden, Germany) and SYBR^TM^ Green Nucleic Acid Stain (Thermo Fisher Scientific Inc., Waltham, MA, USA). Samples were run on a Rotor-Gene Q (QIAGEN, Hilden, Germany) and analyzed with Rotor-Gene Q Series software (QIAGEN, Hilden, Germany).

Primer sequences: β-actin: forward: 5’ TGACAGGATGCAGAAGGAGA 3’, reverse: 5’ CGCTCAGGAGGAGCAATG 3’. IP-10/CXCL10: forward: 5’ GCCGTCATTTTCTGCCTCAT 3’, reverse: 5’ GCTTCCCTATGGCCCTCATT 3’. IFN-β: forward: 5’ CAGGCAACCTTTAAGCATCAG 3’, reverse: 5’ CCTTTGACCTTTCAAATGCAG 3’.

### 2.11. Statistical Analysis 

GraphPad Prism software version 8 (GraphPad Software, San Diego, CA, USA) was used for statistical analysis. The Kruskal–Wallis test followed by Dunn’s post hoc multiple comparisons was used to test for significant differences between multiple groups. The Mann–Whitney U-test was used to test for significant differences between two unpaired groups. Data are shown as median with interquartile ranges. Considered significant were *p* values < 0.05. For the comparison of MF+ mice, Fisher’s exact test was used to test for significant differences. For the comparison of antibody/motility levels over time (Figure 2G–I), a two-way ANOVA with the Geisser–Greenhouse correction and Bonferroni’s (Figure 2F) or Tukey’s multiple-comparisons test (Figure 2G–I) was used. Prior to pooling data from different experiments, data were analyzed for homogeneity by not passing Spearman’s test for heteroscedasticity. If data could not be pooled, but statistical trends (*p* < 0.1) were confirmed by repeated experiments, the trends were indicated in the figure.

## 3. Results

### 3.1. Coimmunization with Poly(I:C) or 3pRNA Enhances Local Immune Responses to Irradiated L3 Larvae 

Based on reports showing that filariae modulate nucleic acid-sensing pathways [44,45,46], we hypothesized that the activation of nucleic acid receptors might enhance worm clearance, and the use of agonists as vaccine adjuvants might increase vaccination efficacy. To that end, the immunostimulatory potential of various nucleic acid receptor agonists was analyzed four hours after subcutaneous injection (Table 1). The injection of all agonists but R848 resulted in increased frequencies of CD11b^+^ cells in the skin. Only the injection of poly(I:C) or 3pRNA, but not R848 or CpG-C, induced a strong local type I IFN response (Table 1). In addition, only the injection of R848 led to a statistically significant increase in systemic IP-10 (*p* = 0.03) (Table 1).

Therefore, poly(I:C) and 3pRNA were selected for further analysis. Poly(I:C) is a known agonist of the RNA sensors TLR3, MDA5, and RIG-I [47,48], while 3pRNA activates RIG-I [49]. Poly(I:C) and 3pRNA were administered as an adjuvant for the immunization with attenuated (att.) *L. sigmodontis* L3 larvae and local as well as systemic immune responses were analyzed four hours after the immunization (Figure 1A). Flow cytometric analysis revealed a significantly increased frequency of neutrophils in the skin after the injection of att. L3 larvae with poly(I:C) or 3pRNA, compared to the 0.9% NaCl control or injection of att. L3 larvae alone (Figure 1B). At the same time, there were significantly decreased frequencies of monocytes and CD11b^+^ DCs in the skins of mice injected with att. L3 larvae and poly(I:C) or 3pRNA, compared to the 0.9% NaCl control or the injection of att. L3 larvae alone (Figure 1C,D). The frequencies of eosinophils were only significantly reduced in mice that received att. L3 larvae in combination with poly(I:C) in comparison to the 0.9% NaCl controls (Figure 1E). Levels of IFN-β and IP-10 (CXCL10) within the skin were increased in response to the injection of att. L3 larvae with poly(I:C) or 3pRNA, compared to the 0.9% NaCl control (Figure 1F,G). In addition, the combination with 3pRNA led to a statistically significant increase of IFN-β (*p* = 0.008) and IP-10 (*p* = 0.02) compared to the injection with att. L3 larvae alone (Figure 1F,G). However, the injection of att. L3 alone did not induce a local IFN-β response. Taken together, poly(I:C) and 3pRNA enhanced local immune responses when coadministered with att. L3 larvae for immunization.

### 3.2. Immunization with Adjuvants Enhances Functional Parasite-Specific Antibody Responses

Since the treatment with poly(I:C) or 3pRNA enhanced local immune responses, the agonists were included in the *L. sigmodontis* immunization strategy. To that end, mice were subcutaneously immunized with att. L3 larvae in combination with poly(I:C) or 3pRNA every two weeks for a total of three times (Figure 2A). This was followed by a natural challenge infection two weeks after the last immunization. The serum obtained two weeks after full immunization, but prior to challenge infection, was analyzed for parasite-specific antibodies. Immunization with att. L3 larvae alone or along with poly(I:C) or 3pRNA induced the production of *L. sigmodontis*-specific IgE compared to nonimmunized mice (Figure 2B). All immunization regimes resulted in significant production of *L. sigmodontis*-specific IgG1 (Figure 2C). The immunization with att. L3 larvae alone did not induce significant levels of *L. sigmodontis*-specific IgG2a/b (Figure 2D). However, there was a significant IgG2a/b response in animals immunized with a combination of att. L3 with poly(I:C) or 3pRNA. The use of poly(I:C) as an adjuvant also significantly increased the IgG2a/b production compared to the immunization with att. L3 larvae alone (Figure 2D). 

In order to assess functionality, an antibody-dependent cellular-cytotoxicity assay was performed. Naïve peritoneal cells, mainly consisting of myeloid and B cells (Figure S1), were cocultured with *L. sigmodontis* L3s, the medium was supplemented with the serum of immunized mice, and the motility of L3s was scored with a five-point scale over time (Figure 2E). The supplementation with a serum of unimmunized mice led to continuous slow motility of L3s (score mean 3.05 ± 0.07 SEM) after one day, and discontinuous movements in part restricted to the ends of the L3s at two and three days of culture (score mean 1.33 ± 0.07 SEM, Figure 2F). The supplementation with serum from mice immunized with att. L3 larvae alone resulted in significantly reduced larvae motilities on days one (score mean 2.40 ± 0.08 SEM) and three (score 0.79 ± 0.14 SEM) of culture. Compared to this, the serum of animals immunized with a combination of att. L3 larvae and poly(I:C) or 3pRNA resulted in significantly reduced larval motility on day one (score 2.07 ± 0.09 for poly(I:C), score 1.81 ± 0.10 for 3pRNA). On day three, only the group with att. L3 and poly(I:C) immunized serum had a motility score that was significantly lower (score 0.42 ± 0.07) compared to the effect of serum from mice immunized with att. L3 alone. 

Parasite-specific antibodies were measured in serum obtained 37, 50, 57, and 63 days after challenge infection. At all times, parasite-specific IgE was lowest in the unimmunized control group (Figure 2G). In all groups, the IgE level remained similar during the infection, with the exception of mice immunized with att. L3 larvae alone, which showed increased IgE values over time with a statistically significant increase from d37 to d50 (*p* = 0.049) and d50 to d63 (*p* = 0.0433). IgG1 levels were lowest in unimmunized mice on day 37 after infection, but highest at the following time points (*p* = 0.0001, *p* < 0.0001, and *p* = 0.0004 at d50, 57, and 63 compared to d37) (Figure 2H). IgG1 levels were mostly similar in all the immunized groups and remained stable during the entire time course with the exception of a statistically significant increase for the att. L3 larvae + poly(I:C) group on day 50 (*p* = 0.0129) and d57 (*p* = 0.0208) in comparison to d37. IgG2a/b levels were lowest in nonimmunized animals (Figure 2I). The immunization with att. L3 larvae alone led to increasing IgG2a/b levels during the course of infection (d37 vs. d63: *p* = 0.0156). Overall, the immunization with att. L3 larvae induced parasite-specific functional antibodies, which were enhanced by the use of nucleic acid-receptor agonists as adjuvants.

### 3.3. Immunization with Poly(I:C) or 3pRNA as Adjuvant Significantly Reduces the Worm Burden Following Challenge Infection in Mice 

Following the serum analysis, we investigated the effect of immunization (Figure 2A) on the adult-worm burden 63 days after the challenge infection (Figure 3A). The immunization with att. L3 larvae alone resulted in a worm burden reduction of 45% compared to unimmunized mice. Poly(I:C) as immunization adjuvant resulted in a significant reduction in worm burden of 73% when compared to unimmunized mice (Kruskal–Wallis test, *p* = 0.0002) and a significant reduction when compared to the immunization with att. L3 larvae alone by direct comparison (Mann–Whitney U test, *p* = 0.019). The use of 3pRNA in combination with att. L3 larvae for immunization resulted in a significant reduction in worm burden by 57% compared to unimmunized animals (Kruskal–Wallis test, *p* = 0.0008). No differences were observed regarding the sex of the worms (Figure S2). 

Of note, none of the immunization regimens led to a complete absence of MF-positive mice (Table 2), indicating that the remaining filariae are viable, fertile, and potentially able to maintain filarial transmission. However, the percentage of MF-positive animals in the control group was 50% and this was reduced to 33.3% by the immunization with att. L3 larvae alone, and further reduced to 29.4% and 16.7% (*p* = 0.043) by the use of poly(I:C) or 3pRNA as the agonist, respectively.

Despite the reduction in worm burden, there were no notable changes in the pleural cell count in any immunized group compared to the control animals (Figure 3B). The concentration of the chemokine CCL11/eotaxin-1 was significantly reduced (*p* = 0.01) in the pleural cavity of mice immunized with a combination of att. L3 larvae and poly(I:C) and reduced by the trend in the pleural cavity of mice immunized with att. L3 larvae and 3pRNA (Figure 3C; *p* = 0.08). The concentration of the chemokine CCL5/RANTES and the proinflammatory cytokine IFN-γ were significantly decreased in the pleural cavities of mice immunized with a combination immunization of att. L3 larvae with poly(I:C) or 3pRNA, compared to unimmunized mice (Figure 3D,E). 

Given that local immune responses in the pleural cavity may be affected by the worm burden, systemic effects, i.e., the composition and cytokine response of spleen cells were additionally quantified. Flow cytometric analysis revealed reduced frequencies of Th1 cells after immunization with 3pRNA and Th17 cells after immunization with poly(I:C) and 3pRNA in the spleen compared to control animals (Figure 3F,H). Frequencies of Th2 and regulatory T cells were similar across all groups (Figure 3G,I). Analysis via ELISA revealed that unstimulated splenocytes from mice immunized with att. L3 alone or a combination with poly(I:C) released significantly higher levels of IL-5 than cells from unimmunized mice (Figure 3J). The IL-5 response to the restimulation with LsAg was significantly enhanced in spleen cells from mice immunized with poly(I:C) in comparison to spleen cells from unimmunized mice. Restimulation with LsAg of the splenocytes from mice immunized with poly(I:C) but not 3pRNA led to a significantly increased release of IP-10 (Figure 3K). At the same time, a significantly increased IFN-γ release by splenocytes from animals immunized with a combination of att. L3 larvae and poly(I:C), compared to cells from unimmunized mice, was detected (Figure 3L). Further, significantly higher IFN-γ responses to the restimulation with LsAg in mice immunized with att. L3 larvae and poly(I:C), compared to the immunization with att. L3 larvae alone were observed. Levels of RANTES were comparable across all groups (Figure 3M).

Taken together, the use of the nucleic acid receptor agonists poly(I:C) or 3pRNA significantly reduced the adult-worm burden, increased parasite-specific immune responses, and enhanced immunization efficacy.

## 4. Discussion

In this study, we demonstrated that the subcutaneous injection of att. L3 larvae along with nucleic acid receptor agonists poly(I:C) and 3pRNA enhanced local immune responses. Furthermore, the implementation of poly(I:C) and 3pRNA as adjuvants during the immunization with irradiated *L. sigmodontis* L3 larvae was successful in enhancing parasite-directed immune responses during subsequent infection, resulting in a reduced adult-worm burden of 73% and 57%, respectively.

Our results demonstrated that nucleic acid receptor agonists such as poly(I:C) and 3pRNA improve the efficacy of a vaccine against the filarial nematode *L. sigmodontis* and may present a novel strategy to boost the efficacy of helminth vaccines that are currently developed. The agonist poly(I:C) mainly targets the cytosolic RNA sensors MDA5 and RIG-I but is also known to activate the endosomal RNA sensor TLR3 [47,48], while the agonist 3pRNA specifically targets RIG-I [49]. Upon subcutaneous injection of the nucleic acid receptor agonists poly(I:C) or 3pRNA alone, there was a rapid influx of neutrophils. Neutrophils are major effector cells that support the elimination of invading *L. sigmodontis* L3 larvae [50,51]. Therefore, it is likely that due to the treatment with poly(I:C) or 3pRNA, part of the L3 larvae were already eliminated in the subcutaneous tissue. Furthermore, it has been shown that type I IFNs enhance the migration of DCs to the draining lymph node and improve their costimulatory potential [52]. In line with this, local type I IFN responses were triggered upon *L. sigmodontis* infection, and DC frequencies decreased after agonist injection. Therefore, the local activation with poly(I:C) and 3pRNA might enhance the activation of innate but also adaptive immune responses targeting the invading L3 larvae. 

In the context of vaccination with irradiated L3 larvae, poly(I:C) as well as 3pRNA enhanced the efficacy of the immunization. Previous studies showed that the immunization with irradiated *L. sigmodontis* L3 larvae resulted in the generation of parasite-specific antibodies [13], a reduction in the adult-worm burden of around 50% [12,17,53,54], and a reduction of MF-positive animals [13]. In contrast to previous studies that used subcutaneous injections of L3 larvae for the challenge infection, we used a natural infection via the mite vector. Despite this difference, our study observed a reduction in the adult-worm burden of 45% for mice that solely received the immunization with irradiated L3 larvae. Furthermore, the induction of parasite-specific IgG1 and IgG2 antibodies, and the reduction in MF-positive animals, was replicated in our study. The addition of poly(I:C) or 3pRNA to the vaccination regimen with irradiated L3 larvae enhanced the production of IgG2a/b antibodies and may have increased the efficacy of the vaccine by inducing a more balanced Th1/Th2 immune response, which was seen in the cytokine measurements from the splenocyte cultures of mice receiving the immunization with poly(I:C) and irradiated L3 larvae. Enhanced parasite-specific antibody levels and differences in isotype switching may also explain that the serum of mice immunized with a combination therapy had superior performance in inhibiting larval motility in the ADCC in vitro assay. In vivo, the immunization with irradiated L3 larvae plus poly(I:C) or 3pRNA further reduced the frequency of MF-positive mice in comparison to the conventional immunization with att. L3 larvae alone. This result is of major importance, as it limits the transmission of the infection. Most importantly, the implementation of the nucleic acid receptor agonists improved vaccination efficacy, as the reduction in the adult-worm burden was most prominent in mice immunized with a combination therapy of att. L3 larvae and poly(I:C) or 3pRNA, reaching a reduction of 73% and 57%, respectively. A similar beneficial impact of nucleic acid receptor agonists on vaccine efficacy was shown for *Schistosoma*, where the activation of the nucleic acid receptors TLR7/8 and TLR9 enhanced the immunization against *Schistosoma japonicum* [55]. Similar to our results, their study observed enhanced production of Th-1-associated cytokines like IFN-γ, and the authors suggested that this contributes to reduced immunomodulation by regulatory T cells. 

Overall, the presented results indicate that type I IFNs may be protective during filarial infection, and targeting the nucleic acids receptors TLR3, RIG-I, and MDA5 by their agonists poly(I:C) and 3pRNA can enhance vaccination efficacy by strengthening protective immune responses and present alternative adjuvants in the context of filarial vaccinations**.**

## Figures and Tables

**Table 1:** Prescreening of potential adjuvants. Mice were injected with poly(I:C), 3pRNA, R848, or CpG-C ODN2395. Skin cells were isolated after 4 h and analyzed for the frequency of CD11b^+^ cells (CD45^+^CD11b^+^) among all leukocytes and the expression of CD86 as well as Δct values of IFN-β expression normalized for β-actin. Additionally, serum IP-10/CXCL10 levels are shown. The median of *n* = 3–4 is shown. Data were analyzed with Kruskal–Wallis and Dunn’s post hoc test.

Injection	CD11b^+^ Cells [%]	CD86 Expression of CD11b^+^ Cells [GMFI]	Local IFN-β Response [Δct]	Systemic IP-10 Response [pg/mL]
0.9% NaCl	60.95	19,563	17.13	348
poly(I:C)	76.1	25,839	8.14	1795
3pRNA	80.7 (*p* = 0.09)	9041	5.49 (*p* = 0.03)	31
R848	65.8	23,483	15.26	5241 (*p* = 0.03)
CpG-C ODN2395	73.8	20,351	15.92	0

**Table 2:** Immunization including poly(I:C) or 3pRNA adjuvant reduces the percentage of animals developing microfilaremia. Mice were immunized three times in two-week intervals by subcutaneous injection of attenuated (att.) *L. sigmodontis* L3 larvae in combination with poly(I:C) or 3pRNA. Two weeks after the last injection, the mice were naturally infected with *L. sigmodontis*. 63 days after the infection microfilariae (MF) were quantified in the peripheral blood. The percentage of MF-positive animals per group was determined, *n* = 6–20. Pooled data from two individual experiments. Statistical significance was assessed via Fisher’s exact test.

Group	Animals Total [#]	MF^+^ Animals [#]	MF^+^ Animals [%]
Control	20	10	50%
att. L3	18	6	33.3% (*p* = 0.342)
att. L3 + poly (I:C)	17	5	29.4% (*p* = 0.315)
att. L3 + 3pRNA	18	3	16.7% (*p* = 0.043)

## Data Availability

The original contributions presented in the study are included in the article/supplementary material and further inquiries can be directed to the corresponding author.

## Supplementary Materials

The following supporting information can be downloaded at: https://www.mdpi.com/article/10.3390/vaccines11050966/s1, Figure S1: Gating strategy of peritoneal exudate cells; Figure S2: Immunization-induced worm clearance affects female and male *L. sigmodontis* filariae similarly.
