# Supplementary material for: Potential of Nucleic Acid Receptor Ligands to Improve Vaccination Efficacy against the Filarial Nematode Litomosoides sigmodontis
Source: Vaccines (Basel). 2023 May 10;11(5):966. doi: 10.3390/vaccines11050966 (PMC10223226; doi:10.3390/vaccines11050966)
Supplement: Supplementary file 1 [file vaccines-11-00966-s001.zip › vaccines-2373384-supplementary.pdf]

A

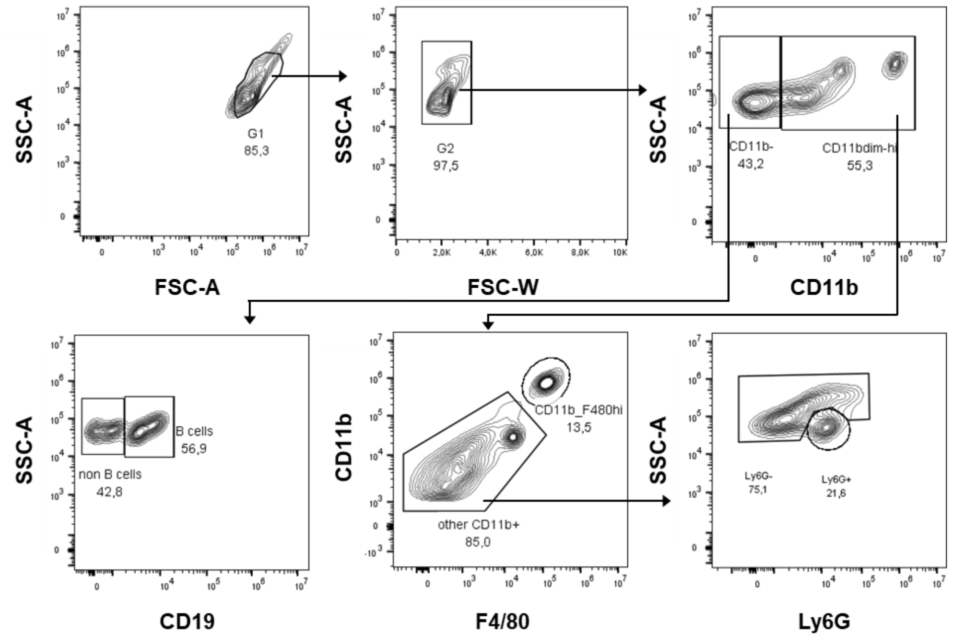

B

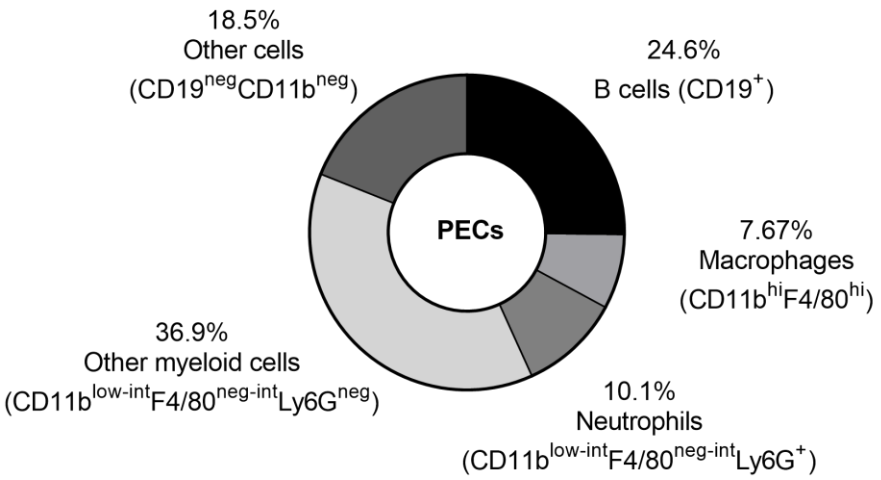

S1 Figure: Gating strategy of peritoneal exudate cells. (A +B) Peritoneal exudate cells were isolated from the peritoneum of naïve BALB/c mice and analyzed by flow cytometry. **(A)** Gating strategy. **(B)** Percentage of B cells, macrophages, neutrophils, other myeloid cell populations (CD11b$^{low\text{-}int}$F4/80$^{neg\text{-}int}$Ly6G^{neg}) and remaining CD19^{-}CD11b^{-} cells. Data from one experiment, representative for three individual experiments.

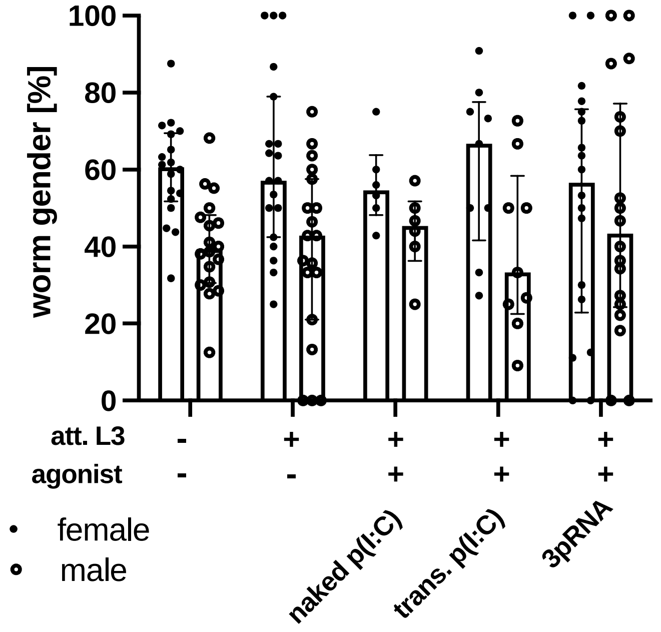

S2 Figure: Immunization-induced worm clearance affects female and male *L. sigmodontis* filariae similarly. Mice were immunized for three times in two-week intervals by subcutaneous injection of attenuated (att.) *L. sigmodontis* L3 larvae in combination with non-formulated poly(I:C) (p(I:C)), p(I:C) or 3pRNA. Two weeks after the last injection, the mice were naturally infected with *L. sigmodontis* for 63 days. Worms were isolated from the pleural cavity and frequencies of females and males were quantified. Error bars show median with IQR. Data were statistically analyzed by 2-way ANOVA with Bonferroni's multiple comparison test. n=6-20. Pooled data from two individual experiments expect for non-formulated poly(I:C).
